# TT2013 meeting report: the Transgenic Technology meeting visits Asia for the first time

**DOI:** 10.1007/s11248-013-9710-y

**Published:** 2013-04-19

**Authors:** Douglas Strathdee, C. Bruce A. Whitelaw

**Affiliations:** 1 Beatson Institute for Cancer Research, Garscube Estate, Switchback Road, Glasgow, G61 1BD UK; 2 Division of Developmental Biology, The Roslin Institute and Royal (Dick) School of Veterinary Studies, University of Edinburgh, Roslin, Midlothian, EH25 9PS UK

**Keywords:** TT2103, China, Transgenic Technology meeting, Embryonic stem cells, ZFNs, TALENs

## Abstract

The 11th Transgenic Technology meeting was held in Guangzhou, China on 25th–27th February 2013. Over 300 scientists and students from round the world gathered to hear the latest developments in the technologies underpinning the creation of transgenic and knockout animals and their application to biological sciences in areas such as the modeling human diseases and biotechnology. As well as informative presentations from leading researchers in the field, an excellent selection of short talks selected from abstracts and posters, attendees were also treated to an inspiring talk from Allan Bradley who was awarded the 9th International Society of Transgenic Technologies Prize for outstanding contributions to the field of transgenic technologies.

Shortly after the 2013 Spring Festival welcoming in the Year of the Snake, the 11th Transgenic Technology meeting took place in Guangzhou, China. It is the first time since the meeting began is Stockholm, Sweden in 1999 that it has visited Asia. We were very warmly welcomed by our Chinese colleagues, who organised the meeting on behalf of the International Society of Transgenic Technologies (ISTT; www.transtechsociety.org). Over 340 participants representing 29 countries gathered in the Baiyun International Convention Center, located in the shadow of White Cloud Mountain in the North of Guangzhou.

The meeting provided a well judged program combining innovative advances in the field as we as practical applications of the technology and providing a rewarding experience for delegates with differing backgrounds. Over 3 days, the sessions covered a wide variety of subjects such as welfare; cryopreservation; epigenetics; functional genomics; embryonic stem (ES) cells and targeted nucleases. In addition, there was the now traditional round table discussion on ‘Aspects of Running a Transgenic Facility’. A couple of highlights among many were the award of the 9th ISTT prize to Allan Bradley (Wellcome Trust Sanger Institute, Hinxton, UK) and the 2nd ISTT Young Investigator Award to Toru Takeo (Division of Reproductive Engineering, Center for Animal Resources and Development-CARD, Kumamoto University, Japan). In addition a number of presentations touched on the growing impact of the innovative targeted nucleases in the generation of transgenics, especially in large animals. Another theme running throughout the meeting was enormous progress and investment made by our Chinese colleagues in the areas of animal transgenesis and functional genomics.

Following the meeting there was a hands on practical work workshop held at the Sun Yat-sen University, Guangzhou. The workshop covered a range of fundamental techniques underlying the generation of transgenic mouse lines organised by Wenhao Xu (University of Virginia, Charlottesville, VA, USA; Chair), Ming Zhao (Southern Medical University, Guangzhou, China), Jing An (Cancer Institute, Southern Medical University, Guangzhou, China) and Liangping Li (Sun Yat-sen University, Guangzhou, China).

After the welcome address from Ming Zhao (Southern Medical University, Guangzhou, PR China; Chair of the TT2013 meeting) and Lluis Montoliu (National Center of Biotechnology, CSIC, Madrid, Spain; ISTT President), the meeting started appropriately enough by a former ISTT prize winner, Qi Zhou (The State Key Laboratory of Reproductive Biology, Institute of Zoology, Chinese Academy of Sciences, Beijing, PR China), the recipient of the 3rd ISTT prize, who detailed his impressive achievements since winning the prize. These included the generation of stem cell derived mice and the generation of mice from haploid ES cells. Jinsong Li (Institute of Biochemistry and Cell Biology, Chinese Academy of Sciences, Shanghai, PR China) then outlined the use of haploid ES cells to produce semi-cloned mice. This significant achievement represents a novel method of generating mouse lines by using a stem cell to fertilise an injected oocyte. In the final talk of the first session Naomi Nagata (Center for Animal Resources and Development, Kumamoto University, Japan) presented an overview of the remarkable progress made in the field of sperm cryopreservation and in vitro fertilization (IVF), a field in which his lab has been at the forefront throughout.

The second session focused on high throughput gene targeting and functional genomics initiatives. Xiang Gao (Model Animal Research Center of Nanjing University, Nanjing, PR China) outlining the impressive progress made in his institution in the generation and phenotyping of genetically altered mouse lines, heralding ‘a new era for mutagenesis in China.’ Michael Dobbie (Australian Phenomics Facility, The Australian National University, Canberra, Australia) gave an overview of the Australian Phenomics Network the creation and characterisation. He also outlined their forward thinking approach combining *N*-ethyl-*N*-nitrosourea (ENU) mutagenesis and next-generation exome sequencing to rapidly identify and exploit ENU alleles of genes of interest. Kent Lloyd (University of California, Davis, USA) gave an overview of the data generated and collated from users of the Knockout Mouse Project (KOMP). Differences in the efficiencies of a variety of different ES cell lines in chimera generation and germline transmission rates were discussed. In addition the advantages of culturing embryonic stem cells in medium containing Map2k1 and Gsk3b inhibitors (2i) were outlined.

Following lunch the first session of the afternoon, focused on what proved to be a recurrent theme throughout the meeting, the application of targeted nucleases to the generation of transgenic animals. Although zinc-finger nucleases (ZFNs) and especially transcription activator-like effector nucleases (TALENs) are a relatively newly developed technology these tools are already making a significant impact in improving the efficiency with which precise genetic modifications can be generated in an enormous variety of species. This session reflected that with three talks all on different species; mice, zebrafish and pigs. Dietmar Kappes (Fox Chase Cancer Center, Philadelphia, PA, USA) firstly illustrated the approach using ZFNs in mice. Using ZFNs his group were able to make numerous precise modifications to the promoter of the Zbtb7b gene, a transcription factor critical for the development of helper T cells. Bo Zhang (College of Life Sciences, Peking University, Beijing, PR China) then discussed the relative ease with which TALENs can be designed and generated. She then went on to illustrate their use in a variety of applications in genome modification in zebrafish. This included heritable gene targeting and the generation of large deletions in the zebrafish genome. Liangxue Lai (Guangzhou Institutes of Biomedicine and Health, Chinese Academy of Sciences, Guangzhou, PR China) then illustrated the use of ZFNs for generation of a PPAR-g gene knockout in pigs. After modification of the gene in fibroblasts, heterozygous animals were generated by nuclear transfer. One note of caution using this technology was that the group also found mutations in 2 of 37 predicted off-target sites.

Next up was the popular round table discussion on ‘How to run a transgenic unit.’ After an introduction from the session chair Jan Parker-Thornburg (MD Anderson Cancer Center, Houston, TX, USA), the first talk was given by Xin-an Pu (The Ohio State Univ., Comprehensive Cancer Center, Columbus, OH, USA) on trouble shooting, what can go wrong with a variety of the techniques underpinning the generation of transgenic mice, such as superovulation, rederivation and DNA preparation. Xin-an then described what to do when things go wrong and how to avoid some common pitfalls. Benoît Kanzler (Max-Planck Inst. for Immunobiology and Epigenetics, Freiburg, Germany) gave an excellent presentation on the very challenging subject of how to develop cutting edge technologies while maintaining an excellent basic service. Central to Benoît’s ideas, were creating the right environment to foster new ideas and having the organization and desire to push new ideas thorough to completion. These are key aspects to help facilitate the development of new technologies. Thomas Kolbe (Biomodels Austria & Inst. For Biotech. in Animal Production, Tulln, Austria) then outlined the factors that you require to take into account when running a transgenic unit as a business.

After the conclusion of the stimulating discussion, Lluís Montoliu (National Center of Biotechnology, CSIC, Madrid, Spain; ISTT President) and Yacine Cherifi (genOway, Lyon, France) presented the 9th ISTT Prize for outstanding contributions to transgenic technologies to Allan Bradley (Wellcome Trust Sanger Institute, Hinxton, UK). During the subsequent Prize Lecture, Allan gave a personal account of his role in the development and application of embryonic stem cells. After Martin Evans had pointed him in the direction of the lab and suggested he should ‘do something useful,’ Allan described how he set about doing exactly that! From ‘not even knowing what a stem cell was’ through developing his own electroporator which required a key and pair of welly boots, Allan described his role at the forefront of development of technology that literally revolutionized functional genomics in the mouse. This involved the development of numerous techniques and reagents which are so widely used today that they can be called standard practice, to his role in developing and overseeing the mouse genome sequencing and characterisation and the development of high throughput gene targeting technology. Together these have facilitated the extraordinary achievement of targeting the vast majority of genes in mouse, a embryonic stem cells, a resource which will allow the study of gene function in a way that was virtually unthinkable, even a few years ago.

The first session of the second day was dedicated mainly to assisted reproduction strategies in mice. Before concentrating on that topic the first speaker, Depei Liu (Chinese Academy of Medical Science and Peking Union Medical College, Beijing, PR China) gave an insight into the use of mouse models in cardiovascular research. In particular the atheroprotective role of Sirt1 was discussed, and its mechanism of action through repression of Shc1, Ccnd1 and Mmp9. Fernanado Benavides (MD Anderson CC, Smithville, TX, USA) gave an illustration of the highly important subject of genetic background and how that can influence the outcome of transgenic experiments. In particular a number of commonly used inbred mouse strains carry specific mutation which can influence the phenotype of interest.

Shannon Byers (The Jackson Laboratory, Bar Harbor, ME, USA) then discussed the advantages of being able to use cryopreserved embryos for blastocyst injection, as well as the development of the ‘perfect host’ strain. These animals are rendered sterile by expression of DTA in the germ cells of the testis, allowing the ES cells space to colonize. Consequently any offspring they produce must be ES cell derived. Francina Langa (Institut Pasteur, Paris, France) presented an assessment of the efficiency of using frozen embryos for blastocyst injection. Although these do work, the efficiency of live birth and chimeras is significantly reduced. Consequently it is necessary to weigh up the convenience of using frozen embryos against the reduction of efficiency in the setting of your particular experiment. Gonzalo Moreno (Instituto de Neurociencias de Alicante, CSIC/UMH, Alicante, Spain) then outlined methodology he had developed allowing the serial surgical collection of sperm samples from mice, without significantly affecting their fertility.

The sixth session at the meeting was dedicated to discussion of ethics and welfare. Malcolm France (University of Sydney, Australia) started off the discussion relating the story of Frances Power Cobbe and the lessons we can learn from the history of the animal rights movement. Catheryn O’Brien (The Walter and Eliza Hall Institute, Melbourne, Australia) then talked about health monitoring programs in transgenic facilities, and the need to balance the costs of a monitoring program with the efficiency of pathogen detection. Catheryn also discussed strategies to avoid spread to disease in a colony and the methods that would facilitate detection. James Bussell (Wellcome Trust Sanger Institute, Hinxton, UK) then gave an impressive illustration of how the factors affecting efficiency of the procedures involved in the production and phenotyping of transgenic colonies can have a dramatic effect on the numbers required within a colony. Careful monitoring of performance indicators can be used to optimize productivity, which consequently has a beneficial effect not only on the quality of the science but also the welfare of the animals.

Two short talks selected from the abstract followed. Firstly, Weibke Garrels (Institute of Farm Animal Genetics, Friedrich-Loeffler-Institut, Mariensee, Germany) described the use of transposons to efficiently introduce defined genetic alterations into the pig genome, in the process identifying permissive loci for gene expression and developing antibiotic-free recombination-mediated cassette exchange and the application of these techniques to the analysis of skin transplantation. Guochun Gong (Regeneron Pharmaceuticals, Inc., Tarrytown, NY, USA) then described the development and characterization of a self deleting antibiotic selection cassette combining a sperm specifically expressed Cre recombinase along with the antibiotic selectable marker.

After a break for lunch and an opportunity to view some of the excellent posters the afternoon’s session was dedicated to the effects of epigenetics of mammalian development and the consequences for the generation of transgenics. Alfonso Gutiérrez-Adán (Department on Animal Reproduction, INIA; Madrid, Spain) gave the first talk of the afternoon about the effects of in vitro culture on mammalian embryos, and the consequential lasting effects on phenotypes in the adult. Alfonso described how culture in suboptimal conditions may lead to changes in body weight organ size and fertility of adult mice. Takashi Kohda (Department of Epigenetics, Medical Research Institute, Tokyo Medical and Dental University, Japan) then described the gene expression changes induced by intracytoplasmic sperm injection (ICSI) which produce marked changes in neonatal mice, but which diminish as the mice become adults. Takashi also showed that there is an overlap between gene expression changes produced by ICSI and somatic cell nuclear transfer suggesting a common underlying mechanism. Guo-Liang Xu (Institute of Biochemistry and Cell Biology, Chinese Academy of Sciences, Shanghai, PR China) described his fascinating work on the mechanisms underlying methylation of the paternal genome in zygotes shortly after fertilization. Guo-Liang described the roles of Tet dioxygenases in the modification of 5-methylcytosine and the subsequent base excision and repair by Tdg. This represents a novel pathway for active demethylation of the genome.

Following a short break there followed a General Assembly of the ISTT and then the meeting banquet, where everybody was treated to some fabulous local cuisine and then danced the night away until the wee small hours.

The third day of the meeting began with a session dedicated to generating transgenics in large animals. Ning Li (State Key Laboratories for Agrobiotechnology, China Agricultural University, Beijing, PR China) started the session talking about genetically engineering of large animals in China and the spectacular progress they have made in recent years. This included numerous examples of transgenic and knockout pigs and cattle. Several different strains of transgenic cattle are now going through biosafety analysis in China with a view to bring these to the market in the next few years. Scott Fahrenkrug (Recombinetics, Minneapolis, MN, USA) outlined the remarkable progress in generating transgenic and knockout pigs and cattle using TALENs. In addition to producing single knockouts by introducing these targeted nucleases into pig fibroblasts, it is also possible to combine multiple TALENs and generate knockouts at two separate alleles simultaneously. Scott also explained that one important and significant advantage of using TALENs is the ability to introduce naturally existing alleles with no requirement to introduce a selectable marker without any footprint. Shoukhrat Mitalipov (Oregon National Primate Res. Center, OHSU, Beaverton, OR, USA) then described the difficulties in generating and validating primate ES cells and the progress made toward a treatment for mitochondrial diseases using a spindle transfer protocol developed in his lab.

Following the session on non-rodent transgenesis, the ninth session of the meeting was devoted to resources available for analysis of transgenic rodents. Yann Herault (Institut Clinique de la Souris, ICS and IGBMC, Illkirch/Strasbourg, France) started off giving an impressive overview of Cre mouse line resources available and where to get access to these. As well as an introduction to the technology Yann described the various repositories available, and the efforts to generate a CreER^T2^ resource. Takashi Kuramoto (Institute of Laboratory Animals, Kyoto University, Kyoto, Japan) gave the next presentation in the session describing the Japanese National BioResource Project for the Rat. This project includes the bio-banking of a variety of rat strains as well as the Rat Phenome Project. One example Takashi described was the development of a colon cancer model, using a point mutant allele of Apc, with one significant advantage of a rat model being the possibility to use endoscopic observation and even the ability to biopsy the tumours in situ.

The penultimate session of the meeting was devoted to applications of ES cell technology. Zhu-Gang Wang (Shanghai Research Center for Model Organisms, Shanghai, PR China) described the mouse mutagenesis program at the Shanghai Research Center. Zhu-Gang then went on to demonstrate that by generating and analysing knockouts of genes with unknown function they were able to come full circle and show that some of these, such as Prss37, a gene the group discovered to be involved in fertility, may play an important role underlying human disease. Masaru Okabe (Genome Information Research Center Research, Institute for Microbial Diseases, Osaka University, Osaka, Japan) demonstrated the progress that his group had made toward differentiation of stem cells into organs. Using rat/mouse interspecies chimeras the group had differentiated rat ES cells into a thymus in a mouse host and then successfully transplanted it back into rats restoring some function. Pentao Liu (Wellcome Trust Sanger Institute, Hinxton, UK) described the important work his group has done in defining additional genes important for improving the efficiency of generating induced pluripotential stem cells (iPSCs). In addition Pentao described the use of TALE transcription factor directed to activate the Oct4 locus could be used to reprogram mouse embryonic fibroblasts into iPSCs. Furthermore Pentao described that using this technology on human cells results in iPSCs which are mouse like mouse ES cells than iPSCs generated by conventional technology.

The final session of the meeting began with the presentation of the 2nd ISTT young investigator award. Ailan Lu (inGenious Targeting Laboratory, Inc., Ronkonkoma, New York, USA) and Lluís Montoliu (National Center of Biotechnology, CSIC, Madrid, Spain; ISTT President) introduced the recipient, Toru Takeo (Division of Reproductive Engineering, Center for Animal Resources and Development-CARD, Kumamoto University, Japan) who received the award for his work in improving mouse sperm cryopreservation and IVF. Toru then presented his award lecture demonstrating the spectacular improvements that he had developed in those areas. At the conclusion of his excellent presentation prizes for the best posters presented at the meeting were awarded to: Da-Wei Yu (State Key Laboratories for Agrobiotechnology, China Agricultural University, Beijing, PR China) for his poster ‘*Expression of Intracellular interferon*-*α in cloned transgenic cattle*’; Ben Davies (Wellcome Trust Centre for Human Genetics, Oxford, UK) for his poster ‘*High efficiency sequence specific mutagenesis on CD1, C57Bl6/J and C3H genetic backgrounds by microinjection of TAL*-*Effector nucleases (TALENs) mRNA*’ and Wojtek Auerbach (Regeneron Pharmaceuticals, Inc., Tarrytown, NY, USA) for his poster ‘*Generation of fully fertile F0 XY female mice from XY ES cells by manipulation of ES cell growth conditions*.’

All in all the 11th Transgenic Technology Meeting proved to be a real success and will be a hard act to follow. A very well done to Ming Zhao and the rest of his organising team who were excellent hosts, making everyone feel extremely welcome. The meeting provided the chance to see some excellent presentations at the forefront of the latest innovations on transgenic technologies. Moreover, in addition to excellent science there were plenty of opportunities to catch up with old friends and also to make new ones. This is what these meetings are all about. The next opportunity for such a get together will again be in 18 months time in October 2014. For its next meeting the ISTT has chosen to come back to Europe and for the first time to the historic city of Edinburgh, Scotland UK. The city of Edinburgh has a rich scientific history from Charles Darwin and James Maxwell to Peter Higgs and Ian Wilmut; the atmospheric cobbled streets of the Old Town will provide a spectacular backdrop to the presentation of the newest innovations in transgenics. We are both very much looking forward to welcoming you all to Edinburgh next year!

